# A Novel Peripheral Action of PICK1 Inhibition in Inflammatory Pain

**DOI:** 10.3389/fncel.2021.750902

**Published:** 2021-12-15

**Authors:** Kathrine Louise Jensen, Gith Noes-Holt, Andreas Toft Sørensen, Kenneth Lindegaard Madsen

**Affiliations:** Molecular Neuropharmacology and Genetics Laboratory, Department of Neuroscience, Faculty of Health and Medical Sciences, University of Copenhagen, Copenhagen, Denmark

**Keywords:** peptide inhibitor, neuropathic pain, PICK1 inhibitor, bivalent peptide, PDZ inhibitor, avidity

## Abstract

Chronic pain is a major healthcare problem that impacts one in five adults across the globe. Current treatment is compromised by dose-limiting side effects including drowsiness, apathy, fatigue, loss of ability to function socially and professionally as well as a high abuse liability. Most of these side effects result from broad suppression of excitatory neurotransmission. Chronic pain states are associated with specific changes in the efficacy of synaptic transmission in the pain pathways leading to amplification of non-noxious stimuli and spontaneous pain. Consequently, a reversal of these specific changes may pave the way for the development of efficacious pain treatment with fewer side effects. We have recently described a high-affinity, bivalent peptide TAT-P_4_-(C5)_2_, enabling efficient targeting of the neuronal scaffold protein, PICK1, a key protein in mediating chronic pain sensitization. In the present study, we demonstrate that in an inflammatory pain model, the peptide does not only relieve mechanical allodynia by targeting PICK1 involved in central sensitization, but also by peripheral actions in the inflamed paw. Further, we assess the effects of the peptide on novelty-induced locomotor activity, abuse liability, and memory performance without identifying significant side effects.

## Introduction

Worldwide, 1.5 billion people are suffering from chronic pain (Goldberg and McGee, 2011). The treatment strategies available leave the majority of these people in more or less constant pain (Goldberg and McGee, 2011; Mills et al., 2019), in part due to the low efficacy of pain relief with numbers needed to treat (NNT) ranging from 6 to 10 (Finnerup et al., 2015; Reinecke et al., 2015; Mills et al., 2019). In addition to these daunting numbers, opioid-based treatment strategies entail a significant risk of inducing addiction (Kaye et al., 2017a, b). Thus, the need for new and better treatments is clear and urgent.

In the ICD-11, chronic pain (MG30) is defined as a multifactorial syndrome of pain that persists for longer than 3 months with biological, psychological, and social factors contributing to the syndrome (WHO, 2021). Chronic pain conditions are characterized by neuropathic as well as inflammatory aspects (Xu and Yaksh, 2011) and while the initial damage is often peripheral (with notable exceptions being traumatic central insults), chronic pain involves central plasticity both in the spinal cord and supraspinal areas (Hartmann et al., 2004; Nagy et al., 2004; Gangadharan et al., 2011; Tan and Kuner, 2021).

For mild to moderate pain, including inflammatory pain conditions, first-line treatment is Non-Steroid Anti-inflammatory Drugs (NSAIDs) and corticosteroids with peripheral action (Varrassi et al., 2019), whereas first-line medication is used to treat neuropathic pain, including anti-convulsant and antidepressant drugs, act by central mechanisms, predominantly in the spinal cord (O’Connor and Dworkin, 2009; Fornasari, 2017). Opioids, on the other hand, are second line treatments that have both peripheral actions as well as central actions at the level of the spinal cord and supraspinal regions (Yaksh and Rudy, 1976; Ferreira and Nakamura, 1979; Rodrigues and Duarte, 2000; Fornasari, 2017). Third-line treatment, includes a combination of medications with peripheral action, such as gabapentin and capsaicin, together with medications with central mechanisms of action (O’Connor and Dworkin, 2009; Varrassi et al., 2019).

An alternative central approach involves targeting glutamatergic transmission in the spinal cord, and drugs targeting NMDA receptors (such as memantine) and AMPA receptors (such as perampanel) are approved for pain treatment. However, since all excitatory communication between neurons depends so heavily on glutamatergic transmission, these drugs are limited by severe side effects including psychosis and coma (Liu and Salter, 2010; Tymianski, 2014). Exploiting the increasing knowledge available on the mechanisms behind dynamic regulation of glutamate receptor function and signaling has led to novel ways for more selective targeting of glutamate receptors leading to pain hypersensitivity (Liu and Salter, 2010; Christensen et al., 2020). Instead of targeting the receptors directly, one can target synaptic scaffold proteins responsible for dynamically regulating the surface expression and ion conductance of the receptors.

An attractive scaffold protein in this context is PICK1 (Protein Interacting with C-Kinase 1). Based on different approaches and methodologies, including the use of siRNA, inhibitory peptides, and knock-out mice, PICK1 has been shown to be implicated in central sensitization of neuropathic pain (Garry et al., 2003; Atianjoh et al., 2010; Wang et al., 2011, 2016). PICK1 is a PDZ domain containing scaffold protein enriched in the postsynaptic density of neurons, known to play a role in synaptic plasticity (Hanley, 2008; Volk et al., 2010). PICK1 is expressed in DRGs and spinal cord in both human and mouse tissue as well as the tibial nerve of humans (Wang et al., 2011; Ray et al., 2018). The PICK1 PDZ domain interacts directly with the C-terminus of a number of different membrane proteins and kinases (Staudinger et al., 1997), including both ASICs and the GluA2 subunit of AMPARs (Dev et al., 1999; Xia et al., 1999; Hu et al., 2010) as well as the transporter responsible for the reuptake of dopamine (DAT) and norepinephrine (NET) (Torres et al., 2001).

We have previously published on a bivalent high-affinity PICK1-inhibitor, TAT-P_4_-(C5)_2_, demonstrating high affinity, good membrane permeability, and low plasma absorption and degradation (Christensen et al., 2020). This peptide has the ability to permeate to the spinal cord following intrathecal (i.t.) injection and access supraspinal areas following i.v. injections (Christensen et al., 2020; Turner et al., 2020). Functionally, TAT-P_4_-(C5)_2_ reduces the interaction between PICK1 and the GluA2-subunit of AMPAR leading to reduced phosphorylation and downregulation of AMPAR surface expression in the spinal cord, thereby alleviating mechanical allodynia in the spared nerve injury (SNI) model of neuropathic pain following i.t. injection (Christensen et al., 2020; Turner et al., 2020).

Whereas intraplantar (i.pl.) administration was insufficient to relieve mechanical allodynia in the SNI model of neuropathic pain in our previous study (Christensen et al., 2020), we found in the present study that TAT-P_4_-(C5)_2_ relieves inflammatory pain by both intraplantar (peripheral) and intrathecal (central) administration.

Given these dual sites of action, systemic administration would be highly attractive to obtain efficacious pain relief. However, since such peptides inevitably need to be membrane permeable to reach the target, they will likely also be present in the CNS. Therefore, it was highly relevant to assess putative central side effect from systemic administration.

Consequently, we assessed putative on- and off-target CNS side effects related to abuse liability, locomotor activity, and memory performance. Together, our data suggest that TAT-P_4_-(C5)_2_ may serve as an attractive lead compound for the development of medications with combined central and peripheral effects in chronic pain conditions with inflammatory aspects, without the dose-limiting side effects or abuse liability seen for opioids.

## Materials and Methods

### Peptide

TAT-P_4_-(C5)_2_ (YGRKKRRQRRR-PEG_4_-(HWLKV)_2_) as HCl salt was ordered from WuXI AppTec (Shanghai, China) with > 95% purity, validated by MS and UPLC.

### Animals

Unless otherwise specified, wild type male C57BL/6NRj mice (Janvier) were used. Animals were allowed at least 7 days of habituation before the start of experiments, which was initiated when the mice were 8 weeks old. Mice were group-housed in a temperature-controlled room, maintained on a 12/12 h light/dark cycle (lights on at 6 A.M.) with free access to standard rodent chow and water. Animal experiments were performed in accordance with guidelines of the Danish Animal Experimentation Inspectorate (permission number 2016-15-0201-00976) in a fully AAALAC-accredited facility under the supervision of the local animal welfare committee.

### Inflammatory Pain

An injury was induced by injection using an insulin needle (0.3 ml BD Micro-Fine) of 50 μl undiluted Complete Freund’s Adjuvant (CFA; F5881, Sigma) unilaterally in the intraplantar surface of the right hind paw, leaving the contralateral left hind paw as an internal control of the pain threshold of the animal. Intraplantar, as well as intrathecal injections were performed while the animal was under isoflurane anesthesia (2%) for maximum of 60 s.

### Mechanical Pain Threshold; von Frey Measurements

Following a minimum of 60 min habituation to the room, the animals were placed in PVC plastic boxes (11.5 cm × 14 cm) on a wire mesh and allowed a minimum of 20 min habituation to the equipment. The mechanical pain threshold of the animals was determined by von Frey measurements (Bioseb, France) on both hind paws. The von Frey filaments used were in the range of 0.04–2 g (g = gram-forces; 0.04, 0.07, 0.16, 0.4, 0.6, 1.0, 1.4, 2.0) and applied to the frontocentral plantar surface of the hind paws. Each filament was applied five times (with adequate resting periods between each application) and the withdrawal threshold was determined as the von Frey filament eliciting at least three out of five positive trials in two consecutive filaments. A positive trial was defined as sudden paw withdrawal, flinching, and/or paw licking induced by a von Frey filament. The baseline mechanical threshold of the animals was measured before pain-induction, and pain-relieving effect of TAT-P_4_-(C5)_2_ was measured on day 2 after pain-induction by either intrathecal (i.t.) or intraplantar (i.pl.) injections of either 20 μM or 200 μM TAT-P_4_-(C5)_2_, 0.9% saline (B.Braun, Germany) or 100 μg/paw morphine hydrochloride (Copenhagen University Hospital Pharmacy, Denmark) all in a volume of 7 μl.

### Locomotion

Animals were habituated to the experimental room for a minimum of 1 h before the initiation of the experiment. At the initiation of the experiment, the mice were injected intraperitoneally (i.p.) with 10 μmol/kg TAT-P_4_-(C5)_2_ (pH adjusted to approximately 7 with 5 M NaOH) or 0.9% saline (B.Braun, Germany) at a volume of 10 μl/g and left in their home cage for 60 min. Mice were then placed in an open field (40 cm × 40 cm × 80 cm) for 60 min with a video camera above, recording their movements.

### Single-Exposure Place Preference

Initial perception of the drugs was measured by single-exposure place preference experiments performed in an elongated three-compartment apparatus (67.5 cm × 24 cm) with a biased design of different floor textures and wall patterns and a neutral zone in the middle. We have previously shown that mice of both genders exhibit a strong bias towards the striped compartment and that a single intraperitoneal exposure of the psychostimulants cocaine and/or amphetamine in the gray compartment is sufficient to change the preference towards that compartment (Runegaard et al., 2017, 2019). For further detail, we refer to Runegaard et al. (2017). Experiments lasted 3 days with exposure sessions on days 1 and 2 and a preference test on day 3. On each day, animals were moved from the housing facility to the experimental room and allowed to acclimatize for at least 60 min before initiation of the experiment. Drug (TAT-P_4_-(C5)_2_/cocaine/gabapentin) was paired with the compartment with holes in the floor and light gray walls, known to be the least preferred compartment (Runegaard et al., 2017, 2019). All mice from a cage were tested at the same time, but not all were given the same treatment. Mice were weighed and injected intraperitoneally with saline, cocaine (10 mg/kg), gabapentin (30 mg/kg) or TAT-P_4_-(C5)_2_ (10 μmol/kg) in a 10 μl/g body weight volume. Depending on the experiment, mice were either placed immediately in the conditioning apparatus after injection or left in their home cage for 60 min before being placed in the conditioning apparatus. The conditioning protocol was counterbalanced so that each animal received one drug injection and one saline injection on either day 1 or day 2 (with the control group receiving saline injections on both days). For the preference test, the plexiglass^®^ partitions between the three compartments were removed and the mice were placed in the neutral zone and allowed to freely explore the apparatus for 20 min. The preference test was recorded, and videos were analyzed with video tracking software (Ethovision XT 13, Noldus) to determine the place preference of the animals. Drug-induced place preference was determined by comparing time spent in the drug-paired compartment between groups (drug vs. saline).

### Spatial Learning and Memory

In the long-term retention experiment, mice were 8–12 weeks old at initiation of the experiment and in the reversal learning experiment all mice were 8 weeks of age at the beginning of the experiment. On each day, the animals were moved from the housing facility to the experimental room and allowed to acclimatize for at least 60 min before initiation of the experiment. All drugs used (0.9% saline or 10 μmol/kg TAT-P_4_-(C5)_2_) were injected intraperitoneally in a 10 μl/g body weight volume. The experiment was divided into Barnes maze habituation, spatial acquisition (training), and probe trials. During *habituation to the Barnes maze*, animals were placed under a cylinder in the middle of the Barnes maze for 10 s and then gently guided to the escape box and left there for 2 min. During *spatial acquisition (training)*, each mouse was placed under a cylinder at the middle of the Barnes maze for 10 s followed by up to 3 min of free exploration of the Barnes maze. Once the animal entered the escape cage, it was left there for 1 min before being transferred back to its home cage for 15–20 min. This training was performed four times per day per mouse. During *probe trials*, the reference memory of the animal was tested by placing each mouse under a cylinder at the middle of the Barnes maze for 10 s followed by 90 s of free exploration of the Barnes maze with no escape cage.

#### Spatial Learning and Memory; Long-Term Retention

Long-term retention was determined by habituation to the Barnes maze on day 1, as well as training on days 1–4. On day 5, all mice were injected intraperitoneally with saline or TAT-P_4_-(C5)_2_ (10 μmol/kg) in a 10 μl/g body weight volume exactly 60 min before being placed on the Barnes maze for their probe trial. On day 12, another probe trial was performed, however with no injection of drugs.

#### Spatial Learning and Memory; Reversal Learning

Reversal learning was determined by habituation to the Barnes maze on day 1, as well as training on days 1–4 and a probe trial on day 5. On days 6–8, the escape hole was moved 90 degrees, and all mice were injected intraperitoneally with saline or TAT-P_4_-(C5)_2_ (10 μmol/kg) in a 10 μl/g body weight volume exactly 60 min before being placed on the Barnes maze for their initial training of the day. On day 9, a second probe trial was performed without any injections, and data analyzed.

### Statistics

All data were analyzed using GraphPad Prism 9.1.1. and presented as mean ±SEM with the significance level set to *p* < 0.05. For locomotion, single exposure place preference, and memory experiments, Ethovision XT 13 (Noldus) was used to track the distance traveled by each animal. For within group analysis (i.e., von Frey experiments) two-way ANOVA followed by Dunnett’s *post hoc* test was used. For between group analysis of multiple time points (i.e., locomotion experiments and training sessions in Barnes maze experiments) two-way ANOVA was used with no *post hoc* test due to lack of treatment-significance in the ANOVA test. When analyzing histograms of only two groups (i.e., saline vs. TAT-P_4_-(C5)_2_ in the learning and memory experiments and locomotion experiment) unpaired t-tests were used when a Gaussian distribution was observed. Otherwise, the unparametric Mann–Whitney test was used. Drug-induced place preference was determined by comparing time spent in the drug-paired compartment between groups (saline vs. drug) with pairwise comparison using a one-way ANOVA followed by Sidak *post hoc* test, if significant ANOVA. The experimenter was blinded to treatment throughout all experiments.

## Results

### Complete Relief of Mechanical Allodynia After Peripheral as Well as Central Administration of TAT-P_4_-(C5)_2_ in Mice

**Figure 1:**
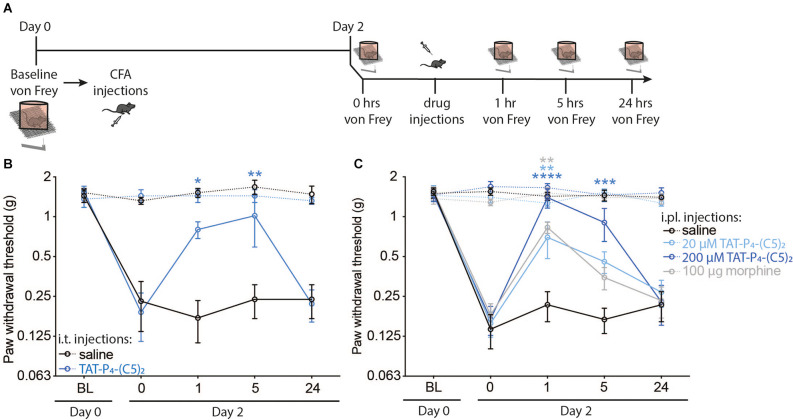
TAT-P_4_-(C5)_2_ reduces acute inflammatory allodynia in mice. *In vivo* experiments revealing the ability of TAT-P_4_-(C5)_2_ to relieve mechanically evoked allodynia in the Complete Freund’s Adjuvant model of inflammatory pain through multiple administration routes. **(A)** Schematic illustration of the timeline and steps of the two experiments assessing the pain-relieving effects of TAT-P_4_-(C5)_2_ in mice. On day 0 a baseline von Frey measurement was followed by an intraplantar injection of CFA into the right hind paw. Hyperalgesia from the CFA injection was confirmed by von Frey measurements on day 2, followed by either central (i.t.) or peripheral (i.pl.) injections of TAT-P_4_-(C5)_2_ or controls. **(B)** I.t. injection of 7 μl 20 μM TAT-P_4_-(C5)_2_ in hyperalgesic animals, leads to significant relief of mechanical allodynia in the injured paw up to 5 h post treatment (TAT-P_4_-(C5)_2_ipsi_; *P*_1 h_ = 0.0434, *p*_5 h_ = 0.0033, *p*_24 h_ = 0.9998, saline__ipsi_; *p*_1 h_ = 0.9963, *P*_5 h_ > 0.9999, *p*_24 h_ > 0.9999, TAT-P_4_-(C5)_2_contra_; *p*_1 h_ > 0.9999, *p*_5 h_ > 0.9999, *p*_24 h_ = 0.9472, saline__contra_; *p*_1 h_ = 0.7556, *p*_5 h_ = 0.2763, *p*_24 h_ 0.8680, Two-way ANOVA followed by Dunnett *post hoc* test of time 0 h vs. 1, 5, 24 h after injection) compared to before injection with no effect on PWT of the contralateral paw or of saline injection (TAT-P_4_-(C5)_2_ipsi_; *p*_1 h_ = 0.0434, *p*_5 h_ = 0.0033, *p*_24 h_ = 0.9998, saline__ipsi_; *p*_1 h_ = 0.9963, *p*_5 h_ > 0.9999, *p*_24 h_ > 0.9999, TAT-P_4_-(C5)_2_contra_; *p*_1 h_ > 0.9999, *p*_5 h_ > 0.9999, *p*_24 h_ = 0.9472, Saline__contra_; *p*_1 h_ = 0.7556, *p*_5 h_ = 0.2763, *p*_24 h_ 0.8680, Two-way ANOVA followed by Dunnett *post hoc* test of time 0 h vs. 1, 5, 24 h after injection. n_saline_ = 5, n _TAT-P4-(C5)*2*_ = 4. **(C)** I.pl. injection of 7 μl 20 μM TAT-P_4_-(C5)_2_ and 200 μM TAT-P_4_-(C5)_2_ or 100 μg morphine all lead to significant relief of mechanical allodynia of the inflamed paw with no effect of injecting saline concentration (20 μM TAT-P_4_-(C5)_2_ipsi_; *p*_1 h_ = 0.0021, *p*_5 h_ = 0.1547, *p*_24 h_ = 0.8732, 200 μM TAT-P_4_-(C5)_2_ipsi_; *p*_1 h_ < 0.0001, *p*_5 h_ = 0.0002, *p*_24 h_ = 0.99913, 100 μg/paw morphine__ipsi_; *p*_1 h_ = 0.0011, *p*_5 h_ = 0.7374, *p*_24 h_ = 0.9948, saline__ipsi_; *p*_1 h_ = 0.9725, *p*_5 h_ = 0.9993, *p*_24 h_ = 0.9725, 20 μM TAT-P_4_-(C5)_2_contra_; *p*_1 h_ = 0.7388, *p*_5 h_ = 0.6524, *p*_24 h_ = 0.5925, 200 μM TAT-P_4_-(C5)_2_contra_; *p*_1 h_ = 0.9991, *p*_5 h_ = 0.4473, *p*_24 h_ = 0.7235, 100 μg/paw morphine__contra_; *p*_1 h_ = 0.3827, *p*_5 h_ = 0.4162, *p*_24 h_ = 0.8861, saline__contra_; *p*_1 h_ = 0.4374, *p*_5 h_ = 0.8561, *p*_24 h_ 0.4176, Two-way ANOVA followed by Dunnett *post hoc* test of time 0 h vs. 1, 5, 24 h after injection). n_saline_ = 8, n_TAT-P4-(C5)2_20 μM_ = 9, n_TAT-P4-(C5)2_200 μM_ = 7, n_morphine_ = 7. *p < 0.05, **p < 0.01, ***p < 0.001, ****p < 0.0001.

Previously, we have shown that TAT-P_4_-(C5)_2_ provides full relief of mechanical allodynia in the spared nerve injury (SNI) model of neuropathic pain following i.t. administration, but not following i.pl. administration (Christensen et al., 2020). In the present study, we tested the effect of TAT-P_4_-(C5)_2_ in the Complete Freund’s adjuvants (CFA)-induced model of inflammatory pain following both i.t. (Figure 1B) or i.pl. (Figure 1C) administration. On day 0, the mechanical paw withdrawal threshold (PWT) was established (PWT of all mice was between 1 and 2 g forces) and the mice were randomly assigned into two (Figure 1B) or four (Figure 1C) different groups. After baseline testing, all animals were injected with 50 μl CFA into the plantar surface of their right hind paw and returned to their home cages (Figure 1A). On day 2, the PWT of the animals was measured and consistent with previous studies (Atianjoh et al., 2010; Aoki et al., 2014), i.pl. injection of CFA led to mechanical allodynia of the ipsilateral paw, with no effect on the contralateral paw; i.e., the gram forces needed to induce paw withdrawal of the ipsilateral hind paw, was significantly lower after CFA injection compared to baseline (Figure 1B; Saline; *p* < 0.0001, TAT-P_4_-(C5)_2_; *p* < 0.0001 ; Figure 1C; Saline; *p* < 0.0001, 20 μM TAT-P_4_-(C5)_2_; *p* < 0.0001, 200 μM TAT-P_4_-(C5)_2_; *p* < 0.0001, morphine; *p* < 0.0001, two-way ANOVA followed by Dunnett *post hoc* test of baseline vs. day 2), which is a behavioral indication of mechanical allodynia. On day 2 we assessed the effect of a single dose of TAT-P_4_-(C5)_2_ administered i.t. under anesthesia (2% isoflurane). Interestingly, 20 μM TAT-P_4_-(C5)_2_ induced a significant increase in the mechanical pain threshold of the injured paw up to 5 h after injection compared to before injection with no effect on PWT of the contralateral paw or of saline injection (TAT-P_4_-(C5)_2_ipsi_; *p*_1 h_ = 0.0434, *p*_5 h_ = 0.0033; Figure 1B). Next, we tested the peripheral effects of two different concentrations of TAT-P_4_-(C5)_2_ by injecting it straight into the inflamed paw and measuring the PWT of both hind paws 1, 5, and 24 h after injection (Figure 1C). As a positive control, we injected 100 μg/paw morphine (Rodrigues and Duarte, 2000), and as a negative control, we injected 0.9% saline solution. Both doses of TAT-P_4_-(C5)_2_ revealed significant reversal of the CFA-induced mechanical allodynia at 1 h post administration, with the highest dose showing relief of mechanical allodynia at 5 h post injection with no effect on the contralateral paw at any time point of either concentration (20 μM TAT-P_4_-(C5)_2_ipsi_; *p*_1 h_ = 0.0021, 200 μM TAT-P_4_-(C5)_2_ipsi_; *p*_1 h_ < 0.0001, *p*_5 h_ = 0.0002; Figure 1C). The positive control (morphine), showed relief of mechanical allodynia at 1 h post injection with no effect of the contralateral paw, and the negative control (saline), showed no effect at any time point on either paw (100 μg/paw morphine__ipsi_; *p*_1 h_ = 0.0011; Figure 1C). Importantly, since we previously demonstrated that i.t. injection but not i.pl. injections of 20 μM nor 200 μM TAT-P_4_-(C5)_2_ significantly relieved mechanical allodynia in the SNI model, we can firmly conclude that the action of the i.pl. injection of TAT-P_4_-(C5)_2_ in the CFA model is indeed peripheral.

### TAT-P_4_-(C5)_2_ Does Not Significantly Affect Basal Locomotion in Mice

**Figure 2:**
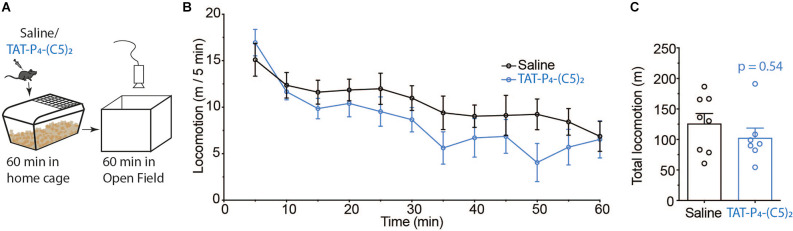
No effect of TAT-P_4_-(C5)_2_ on locomotion. **(A)** Schematic overview of the open field experiment setup used to assess the effect of TAT-P_4_-(C5)_2_ on novelty-induced exploration. **(B,C)** *In vivo* experiments revealing no effect of TAT-P_4_-(C5)_2_ on basal locomotion. Mice were injected intraperitoneally with 0 or 10 μmol/kg TAT-P_4_-(C5)_2_ and left in their home cage for 60 min before being placed in the open field for 60 min. Data is depicted in bins of 5 min **(B)** and as total locomotion **(C)** within the 60 min spent in the open field. No significant different between groups in neither **(B)** (*F*_(1,13)_ = 1.02, *p* = 0.33, two-way repeated measures ANOVA) nor **(C)** (*p* = 0.54, Mann-Whitney t-test). n_saline_ = 8, n_TAT-P_4_-(C5)_2__ = 7. All data are expressed as mean ±SEM.

PICK1 plays an important role in AMPAR related synaptic transmission as well as dopamine homeostasis (Madsen et al., 2005; Xu and Xia, 2006; Jensen et al., 2018). Therefore, we tested if TAT-P_4_-(C5)_2_ affected general locomotion in a classic open field test (Figure 2). Since we know the pain-relieving effects of TAT-P_4_-(C5)_2_ to be efficient at one hour after injection (Christensen et al., 2020; Figure 1), we tested the locomotion of the animals at this time point. Animals were injected i.p. with either 0.9% saline or 10 μmol/kg TAT-P_4_-(C5)_2_ and placed in their home cage for 1 h before being placed in the open field box and their locomotion was monitored for a further 60 min (Figure 2). Although a tendency was seen, locomotion was not reduced (Figure 2B). Also, the total distance traveled during 60 min did not show any difference between treatment groups (Figure 2C).

### TAT-P_4_-(C5)_2_ Shows No Indication of Neither Aversive Nor Addictive Properties During Initial Perception of the Peptide

**Figure 3:**
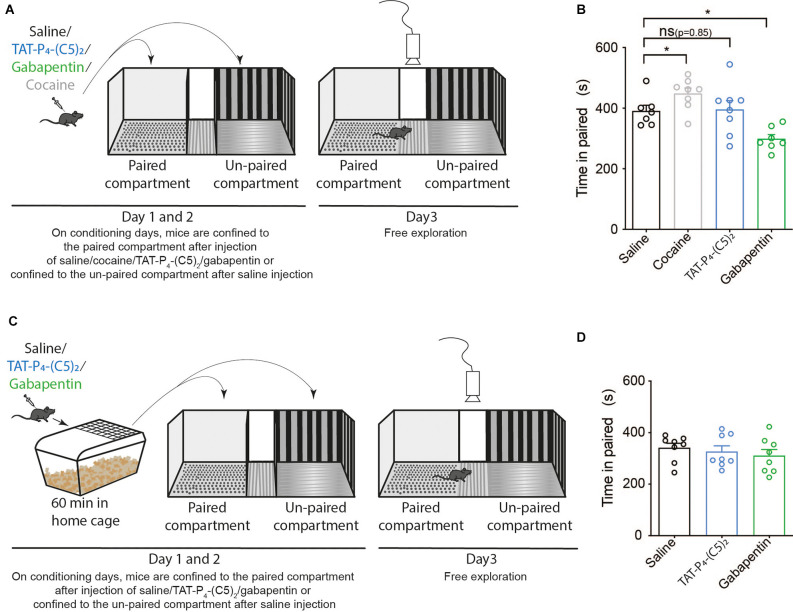
TAT-P_4_-(C5)_2_ show no addictive and aversive properties in mice. TAT-P4-(C5)_2_ shows no indication of neither aversive nor addictive properties during the initial perception of the peptide. **(A)** Schematic overview of a classic single-exposure place preference (sePP) test with two counter-balanced days of conditioning followed by a test day. During conditioning days, mice were injected intraperitoneally (i.p.) with either saline, TAT-P4-(C5)_2_, gabapentin, or cocaine and placed directly in the confined conditioning compartments. The drug was always given before placement in the gray paired compartment and saline before placement in the striped un-paired compartment. On day 3, mice were placed in the unconfined apparatus with free exploration between the three sections and their movement between sectors was recorded. **(B)** The positive control (cocaine) led to a significant increase in time spent in the paired compartment compared to the saline group (Figure 1B; one-way ANOVA; *F*_(3,24)_ = 11.38, *p* < 0.0001, followed by šídák multiple comparison; *p* = 0.02) during the preference test on day 3. TAT-P_4_-(C5)_2_ had no effect on the preference (Figure 1B; one-way ANOVA; *F*_(3,24)_ = 11.38, *p* < 0.0001, followed by šídák multiple comparison; *p* = 0.85). Gabapentin showed aversive effects, decreasing the preference for the paired compartment even further (Figure 1B; one-way ANOVA; *F*_(3,24)_ = 11.38, *p* < 0.0001, followed by šídák multiple comparison; *p* = 0.049). n_saline_ = 7, n_cocaine_ = 8, n_TAT-P_4_-(C5)_2__ = 8, n_gabapentin_ = 7. **(C)** Schematic overview of a modified sePP test with two counter-balanced days of conditioning followed by a test day. During conditioning days, mice were injected i.p. with either saline, TAT-P_4_-(C5)_2_, gabapentin, or cocaine and placed in their home cage for an hour before placement in the confined conditioning compartments. The drug was always paired with the gray compartment (paired), and saline was paired with the striped compartment (unpaired). On day 3, mice were placed in the unconfined apparatus with free exploration between the three sections and their movement between sectors was recorded. **(D)** Neither TAT-P_4_-(C5)_2_ (*F*_(2,21)_ = 0.4999, *p* = 0.61, one-way ANOVA) nor gabapentin (*F*_(2,21)_ = 0.4999, *p* = 0.61, one-way ANOVA) showed any effect on place preference when conditioning took place an hour post injection. n_saline_ = 8, n_TAT-P_4_-(C5)_2__ = 8, n_gabapentin_ = 8. All data are expressed as mean ± SEM. Abbreviations: ns = non-significant, sePP = single exposure place preference. *p < 0.05.

Due to the high abuse liability of current treatments for chronic pain, and in particular opioids (Smith et al., 2016; Gostin et al., 2017), we wanted to assess the abuse liability of TAT-P_4_-(C5)_2_. The initial sensitivity to the rewarding properties of drugs is believed to be an important endophenotype in relation to the vulnerability to addiction (Lambert et al., 2006). The experiment was performed in a three-compartment apparatus with a striped and gray compartment separated by a neutral white zone (Figure 3A). Here, we injected the mice with either the psychostimulant cocaine, our peptide TAT-P_4_-(C5)_2_, or gabapentin (a first-line treatment for chronic pain) and investigated their ability to induce place-preference in this single-exposure set-up (Figure 3A). As expected, cocaine led to a significant increase in time spent in the paired compartment compared to the saline group (Figure 3B; *p* = 0.02) during the preference test on day 3. TAT-P_4_-(C5)_2_ had no effect on the preference (Figure 1B; *p* = 0.85), but surprisingly gabapentin showed aversive effects decreasing the preference for the paired compartment even further (Figure 3B; *p* = 0.049). Since the pharmacokinetics of TAT-P_4_-(C5)_2_ is likely slower than that of cocaine, we repeated the experiment with an hour’s delay between drug injection and placing the animals in the conditioning compartments (Figure 3C). Neither TAT-P_4_-(C5)_2_ nor gabapentin showed any effect on place preference in this setup (Figure 1D).

### TAT-P_4_-(C5)_2_ Shows No Indication of Inducing Memory Side Effects, Neither Long-Term Retention Nor Reversal Learning

PICK1 is required for hippocampal long-term potentiation (LTP) and long-term depression (LTD), cerebellar LTD, Ca^2+^-permeable AMPAR plasticity, and mGluR LTD in the perirhinal cortex (Xia et al., 1999; Gardner et al., 2005; Liu and Cull-Candy, 2005; Steinberg et al., 2006; Terashima et al., 2008; Clem and Huganir, 2010; Thorsen et al., 2010; Volk et al., 2010).

**Figure 4:**
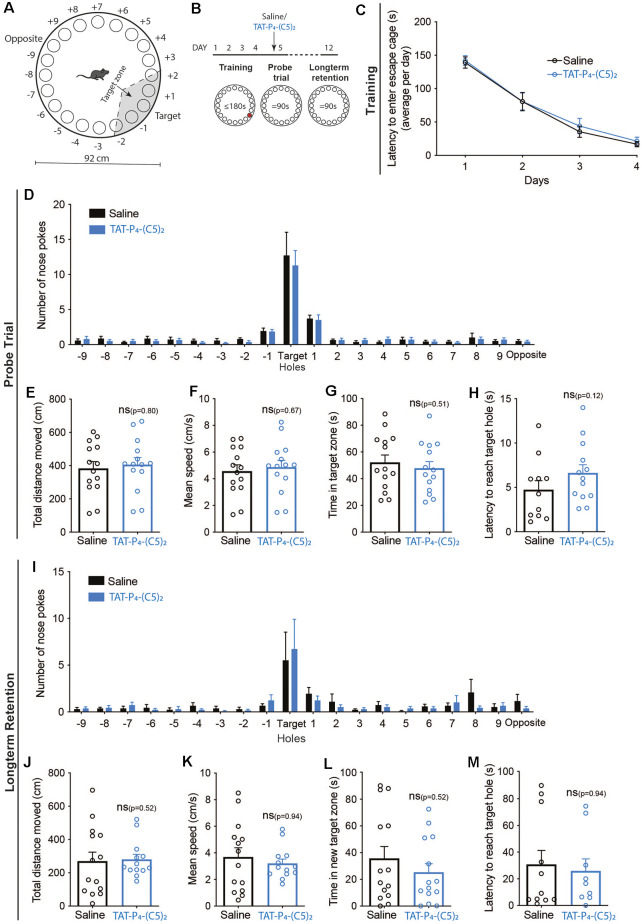
Long-term memory retention is not influenced by TAT-P_4_-(C5)_2_. **(A)** Schematic illustration of the Barnes maze used to assess long-term memory retention of the mice. The Barnes maze is a circular platform of 92 cm in diameter with 20 holes equally spaced around the perimeter edge and a detachable escape cage under one of the 20 holes. **(B)** The long-term retention experiment was comprised of a 12-day protocol with habituation on day 1, training on day 1–4, and probe tests on days 5 and 12. Saline or 10 μmol/kg TAT-P_4_-(C5)_2_ was injected intraperitoneally prior to the probe trial on day 5 and the probe test on day 12 was used to assess the long-term memory retention of the animals. **(C)** Both groups showed a significant decrease in latency to enter the escape cage over the 4 days of training, reflecting their learning and memory of the escape cage location (*F*_time(2.316,60.22)_ = 107.8, *p* < 0.0001, *F*_subject(26,78)_ = 3.756, *p* < 0.0001, two-way ANOVA), with no difference between groups (*F*_group(1,26)_ = 0.1648, *p* = 0.69, two-way ANOVA). **(D)** At the probe test on day 5, no difference was observed between groups in relation to nose pokes (*F*_group(1,520)_ = 0.3882, *p* = 0.534, 2-way ANOVA). **(E)** At the probe test on day 5, no difference was observed between groups in relation to the total distance moved (*p* = 0.80, Mann-Whitney t-test). **(F)** At the probe test on day 5, no difference was observed between groups in relation to the mean speed of the animals (*p* = 0.67, Mann-Whitney t-test). **(G)** At the probe test on day 5, no difference was observed between groups in relation to the time spent in the target zone (*p* = 0.51, Mann-Whitney t-test). **(H)** At the probe test on day 5, no difference was observed between groups in relation to their latency to reach the target hole (*p* = 0.12, Mann-Whitney t-test). **(I)** At the long-term memory retention probe test on day 12, no difference was observed between groups in relation to nose pokes (*F*_group(1,520)_ = 0.1537, *p* = 0.695, 2-way ANOVA). **(J)** At the long-term memory retention probe test on day 12, no difference was observed between groups in relation to the total distance moved (*p* = 0.52, Mann-Whitney t-test). **(K)** At the long-term memory retention probe test on day 12, no difference was observed between groups in relation to the mean speed of the animals (*p* = 0.94, Mann-Whitney t-test). **(L)** At the long-term memory retention probe test on day 12, no difference was observed between groups in relation to the time spent in the target zone (*p* = 0.52, Mann-Whitney t-test). **(M)** At the long-term memory retention probe test on day 12, no difference was observed between groups in relation to the latency to reach the target hole (*p* = 0.94, Mann-Whitney t-test). n_saline_ = 14, n_TAT-P_4_-(C5)_2__ = 14. All data are expressed as mean ± SEM. Abbreviations: ns = non-significant.

**Figure 5:**
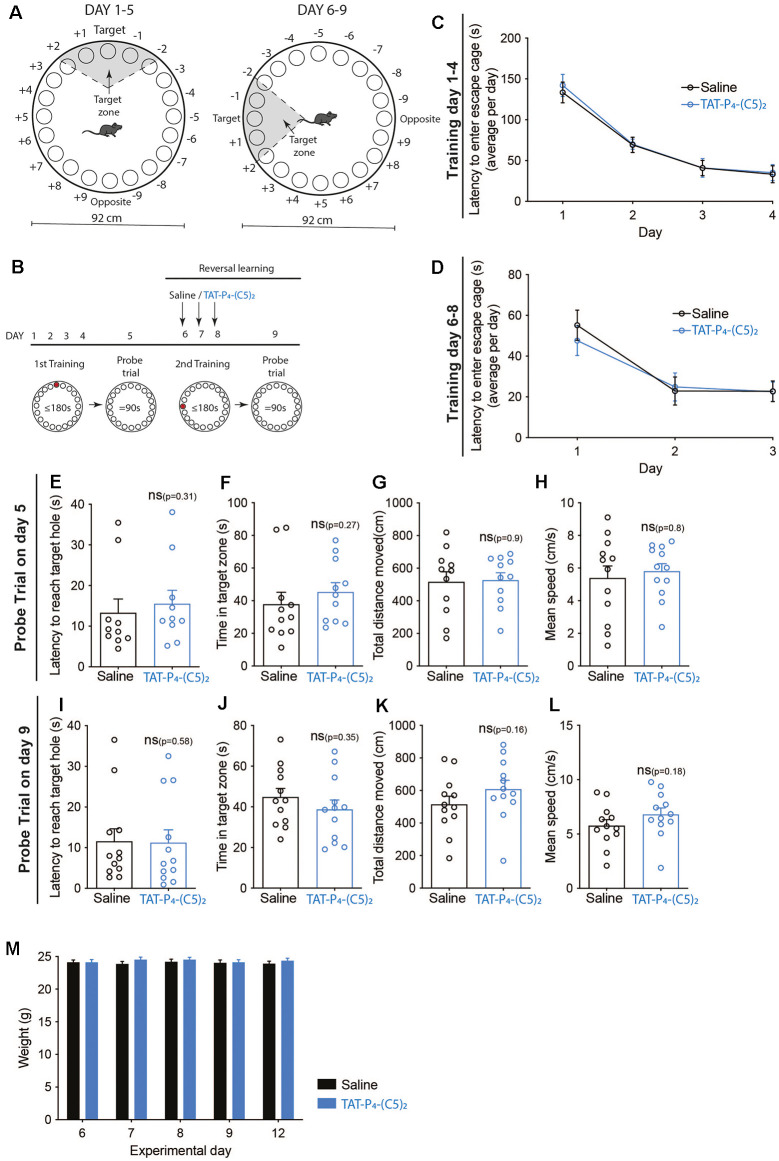
No effect of TAT-P_4_-(C5)_2_ on reversal learning. **(A)** Schematic illustration of the Barnes maze used to assess long-term memory retention of the mice. The Barnes maze is a circular platform of 92 cm in diameter with 20 holes equally spaced around the perimeter edge and a detachable escape cage under one of the 20 holes. To test the reversal learning of the mice, the escape cage was rotated 90 degrees counter-clockwise following the first probe trial. **(B)** The long-term retention experiment was comprised of a 9-day protocol with habituation on day 1, training on day 1–4, and probe tests on day 5. At this point, the escape cage is rotated 90 degrees counter-clockwise, and mice are then injected intraperitoneally with saline or 10 μmol/kg TAT-P_4_-(C5)_2_ depending on their groups on the following three additional training days. On day 9, a second probe trial was performed to assess the reversal learning abilities of the animals dependent on TAT-P_4_-(C5)_2_ injection. **(C)** Both groups showed a significant decrease in latency to enter the escape cage over the 4 days, reflecting their learning and memory of the escape cage location (*F*_time(1.799,37.78)_ = 55.11, *p* < 0.0001, *F*_subject(21,63)_ = 2.493, *p* = 0.0028, two-way ANOVA), with no difference between groups (*F*_group(1,21)_ = 0.0818, *p* = 0.78, two-way ANOVA). **(D)** Both groups showed a significant decrease in latency to enter the rotated escape cage over the three days of the second round of training on days 6–8, reflecting their ability to learn and memorize the new escape cage location (*F*_time(1.462,30.70)_ = 28.09, *p* < 0.0001, *F*_subject(21,42)_ = 4.743, *p* < 0.0001, two-way ANOVA), with no difference between groups (*F*_group(1,21)_ = 0.0634, *p* = 0.80, two-way ANOVA). **(E)** At the probe test on day 5, no difference was observed between groups in relation to the latency to reach the target hole (*p* = 0.31, Mann-Whitney t-test). **(F)** At the probe test on day 5, no difference was observed between groups in relation to the time spent in the target zone (*p* = 0.27, Mann-Whitney t-test). **(G)** At the probe test on day 5, no difference was observed between groups in relation to the total distance moved (*p* = 0.9, Mann-Whitney t-test). **(H)** At the probe test on day 5, no difference was observed between groups in relation to the mean speed of the animals (*p* = 0.8, Mann-Whitney t-test). **(I)** At the reversal learning probe test on day 9, no difference was observed between groups in relation to the latency to reach the target hole (*p* = 0.58, Mann-Whitney t-test). **(J)** At the reversal learning probe test on day 9, no difference was observed between groups in relation to the time spent in the target zone (*p* = 0.35, Mann-Whitney t-test). **(K)** At the reversal learning probe test on day 9, no difference was observed between groups in relation to the total distance moved (*p* = 0.16, Mann-Whitney t-test). **(L)** At the reversal learning probe test on day 9, no difference was observed between groups in relation to the mean speed of the animals (*p* = 0.18, Mann-Whitney t-test). **(M)** On the morning of days 6, 7, 8, 9, and 12 all mice were weighed. No weight-effect on neither time (*F*_time(2.954,62.03)_ = 1.396, *p* = 0.26, two-way ANOVA), nor treatment (*F*_group(1,21)_ = 0.4207, *p* = 0.52, two-way ANOVA) over the duration of the experiment was found. n_saline_ = 12, n_TAT-P_4_-(C5)_2__ = 12. All data are expressed as mean ± SEM. Abbreviations: ns = non-significant.

Consequently, we tested spatial learning and memory performance of mice following intraperitoneal administration of 10 μmol/kg TAT-P_4_-(C5)_2_ (Figures 4, 5) using a Barnes maze (Barnes, 1979; Bach et al., 1995). We used a standard design with a circular platform top of 92 cm in diameter with 20 holes equally spaced around the perimeter edge and a detachable escape cage under one of the 20 holes (Figures 4A, 5A).

In the first set-up, we investigated whether TAT-P_4_-(C5)_2_ affects long-term memory retention (Figure 4). The mice were divided into two groups and went through 4 days of training, learning the location of the escape cage (Figure 4C). Both groups showed a significant decrease in latency to enter the escape cage over the four days, reflecting their learning and memory of the escape cage location (*F*_time(2.316,60.22)_ = 107.8, *p* < 0.0001, *F*_subject(26,78)_ = 3.756, *p* < 0.0001, two-way ANOVA), with no difference between groups. On day 5, the mice were injected with TAT-P_4_-(C5)_2_, and a probe trial was performed (Figures 4D–H). Again, no difference was observed between groups neither in relation to nose pokes (Figure 4D), total distance moved (Figure 4E), mean speed of the animals (Figure 4F), time spent in target zone (Figure 4G), nor in their latency to reach the target hole (Figure 4H). On day 12, their long-term retention was assessed through a second probe trial, and again no difference was observed between the two groups on any of the investigated parameters; nose pokes (Figure 4I), total distance moved (Figure 4J), mean speed of the animals (Figure 4K), time spent in target zone (Figure 4L), or their latency to reach the target hole (Figure 4M).

In the second set-up, we investigated whether TAT-P_4_-(C5)_2_ affects reversal learning (Figure 5) by rotating the location of the escape cage 90 degrees counter-clockwise after the first probe trial, followed by three additional training sessions and ending with a second probe trial (Figure 5B). This time, the mice were injected with TAT-P_4_-(C5)_2_ on days 6, 7 and 8 before initiation of the training session of those days (Figure 5B).

The mice were divided into two groups and went through 4 days of training, learning the initial location of the escape cage (Figure 5C). Both groups showed a significant decrease in latency to enter the escape cage over the 4 days, reflecting their learning and memory of the escape cage location (*F*_time(1.799,37.78)_ = 55.11, *p* < 0.0001, *F*_subject(21,63)_ = 2.493, *p* = 0.0028), with no difference between treatment groups. On day 5, the probe trial was performed (Figures 5E–H). As expected, no difference was observed between groups, neither in relation to latency to reach the target hole (Figure 5E), time spent in target zone (Figure 5F), total distance moved (Figure 5G), or the mean speed of the animals (Figure 5H). After rotating the escape cage 90 degrees counter-clockwise, the mice underwent daily injections of TAT-P_4_-(C5)_2_ followed by new training sessions, for three consecutive days. During the training sessions, TAT-P_4_-(C5)_2_ did not affect the reversal learning of the mice compared to the saline group (Figure 5D). Both groups showed a significant decrease in latency to enter the rotated escape cage over the 3 days, reflecting their ability to learn and memorize the new escape cage location (*F*_time(1.462,30.70)_ = 28.09, *p* < 0.0001, *F*_subject(21,42)_ = 4.743, *p* < 0.0001), with no difference between groups. On day 9, the second probe trial was performed (Figures 5I–L). Again, no difference was observed between groups neither in relation to latency to reach the target hole (Figure 5I), time spent in the target zone (Figure 5J), total distance moved (Figure 5K), or the mean speed of the animals (Figure 5L). Since the animals were injected with TAT-P_4_-(C5)_2_ for 3 days in a row, and PICK1 is involved in metabolism by affecting insulin and growth hormone storage and release (Cao et al., 2013; Holst et al., 2013; Herlo et al., 2018; Li et al., 2018), we monitored their weight (Figure 5M). Weight measurements were performed in the morning, before initiating the experiment on that day, and no effect on either time nor treatment over the duration of the experiment was found.

These results show that TAT-P_4_-(C5)_2_ can be administered systemically to alleviate both peripheral and central sensitization to mechanical allodynia involving inflammatory aspects, without putative on-target side effects on locomotion and memory performance and without apparent abuse liability.

## Discussion

The prevalence of inflammatory pain is high, with about 350 million people worldwide suffering from arthritis and joint disease (Pahwa et al., 2021). In general, diseases associated with chronic inflammation are expected to increase (Pahwa et al., 2021).

Inflammatory pain is caused by the activation and sensitization of nociceptive pain pathways by mediators released at sites of tissue inflammation (Woolf and Salter, 2000; Xu and Yaksh, 2011). In acute inflammatory models, like the CFA model used in this study, pain arises as a result of damage to the tissue, leading to hyperalgesia in that specific area as well as the adjacent normal tissue. The pain typically resolves as the tissue heals. However, the consequence of the CFA injection is not merely at the injection site, since several changes occur in the dorsal root ganglia, as well as the dorsal horn. One of these changes is the strengthened synaptic efficacy of the spinal cord dorsal horn, developing from an enhanced function of postsynaptic glutamate receptors (Woolf and Salter, 2000; Liu and Salter, 2010; Xu and Yaksh, 2011). Both inflammatory and neuropathic pain models involve an upregulation of calcium permeable AMPARs; a type of ionotropic transmembrane glutamate receptor (Vikman et al., 2008; Gangadharan et al., 2011; Chen et al., 2013). PICK1 is involved in the maladaptive expression of these calcium permeable AMPARs in both the midbrain, hippocampus, and the spinal cord (Dixon et al., 2009; Wolf and Ferrario, 2010; Luscher and Malenka, 2011; Christensen et al., 2020). The shared mechanism behind inflammatory and neuropathic pain may argue that TAT-P_4_-(C5)_2_ can alleviate the central aspect of both types of pain. We have previously shown that TAT-P_4_-(C5)_2_ can alleviate mechanical allodynia in the SNI model of neuropathic pain following intrathecal administration, while no effect was observed following peripheral (intraplantar) administration (Christensen et al., 2020). In the present study, we show that TAT-P_4_-(C5)_2_ alleviates pain in the CFA model of inflammatory pain following both central (i.t.) and peripheral (i.pl.) administration of the peptide (Figure 1).

Scaffold proteins belong to a highly diverse family of proteins known to ensure specificity in intracellular signaling networks by orchestrating neuronal signaling processes (Kim and Sheng, 2004; Good et al., 2011). Since PICK1 is involved in regulating surface expression of glutamate receptors and excitatory communication between neurons in the central nervous system almost exclusively rely on glutamatergic neurotransmission, putative side effects of inhibiting PICK1 with the TAT-P_4_-(C5)_2_ peptide is a valid concern.

The role of PICK1 in AMPAR related synaptic transmission, as well as dopamine homeostasis (Madsen et al., 2005; Xu and Xia, 2006; Jensen et al., 2018), could mean that inhibition of PICK1 with TAT-P_4_-(C5)_2_, would affect basal behaviors such as locomotion. PICK1 has a direct interaction with the c-terminus of DAT as well as PKCα, which could give PICK1 a role in the regulation of PKC-evoked DAT trafficking, phosphorylation, and/or function by bringing DAT and PKC in close proximity to each other (Staudinger et al., 1995; Torres et al., 2001). An easy outcome measure of the functionality of DAT, is locomotion since the role of DAT is the re-uptake of extracellular dopamine from the extracellular cleft. Increased striatal dopamine levels lead to hyperlocomotion of mice (Pijnenburg et al., 1976). Concurrently, stimulating either D1 or D2 receptors in the nucleus accumbens leads to increased locomotion, with the synergistic effect of activating both (Gong et al., 1999) and inhibiting DAT directly with systemic administration of e.g., cocaine leads to hyperlocomotion as well (Giros and Caron, 1993). During the exploration of new places, striatal dopamine release leads to increased locomotion in a highly regulated process of momentarily increased extracellular dopamine (Mejias et al., 2021). Temporarily inhibiting PICK1 with TAT-P_4_-(C5)_2_ could theoretically prevent the increased dopamine release, thereby inhibiting the locomotion of the animals. However, we do not see any effect of TAT-P_4_-(C5)_2_ on novelty-induced locomotion (Figure 2), which suggests that either the phenotypic effect of compromised DAT/PKC interaction is relatively modest or that even though the peptide reaches the midbrain (Turner et al., 2020), the concentration is not sufficient to effectively modulate the DAT/PKC regulation. Either way, it demonstrates that systemic administration of TAT-P_4_-(C5)_2_ is compatible with functional dopamine signaling in the midbrain in agreement with our previous findings that i.v. administration of TAT-P_4_-(C5)_2_ neither compromised locomotor activity nor sucrose seeking in rats (Turner et al., 2020).

As mentioned, two equally important problems with the current treatment strategies for chronic pain exist. Firstly, the efficacy of pain relief is very low (Finnerup et al., 2015; Reinecke et al., 2015) and secondly, aggressive marketing of opioids for patients in chronic pain has fueled an opioid epidemic in the United States (Gostin et al., 2017). To get an indication of whether TAT-P_4_-(C5)_2_ shows addictive potential, we performed a single exposure place preference experiment. An increase in extracellular dopamine is implicated in reward-related behavior and addiction and a single dose of the highly addictive psychostimulant cocaine is sufficient to change the AMPAR/NMDAR ratio of the ventral tegmental area of the brain (Ungless et al., 2001). In addition, it is believed that the initial sensitivity to the rewarding properties of drugs is an important indicator of addictive properties (Lambert et al., 2006; Runegaard et al., 2017). From our data (Figure 3), there is no indication, that TAT-P_4_-(C5)_2_ has addictive properties, since the preference for the TAT-P_4_-(C5)_2_-paired compartment is the same as that for saline. However, in order to assess this for successive administrations envisioned for chronic pain treatment, a full-scale conditioned place preference setup or self-administration experiments would need to be performed.

An interesting finding from our sePP experiment is the apparent aversive effect of gabapentin (Figure 3B). This could very likely reflect the side effects seen with gabapentin such as drowsiness, weakness, anxiety, dizziness, headache, and double/blurred vision. Gabapentin was originally developed as an anti-epileptic drug but has since been used off-label to treat a wide array of disorders such as neuropathic pain, insomnia, menopausal conditions, bipolar disorder, drug and alcohol addiction, anxiety, migraine, and more (Smith et al., 2016). Previously gabapentin was presumed not to have addictive properties (Bonnet et al., 1999; Lavigne et al., 2012), but increasing reports on misuse among humans have since started to appear (Smith et al., 2016), which highlights the importance of testing drugs for addictive properties earlier in the drug development process.

Disrupting the interaction between PICK1 and the GluA2 subunit has been shown to alter synaptic plasticity *ex vivo*, a molecular mechanism of learning and memory. *In vivo*, adult PICK1 KO animals show impaired hippocampal-dependent learning and memory, tested by inhibitory avoidance learning (Volk et al., 2010). Because of the putative role of PICK1 in memory, we thoroughly tested whether TAT-P_4_-(C5)_2_ showed any indication of affecting long-term retention as well as reversal learning but saw no indication that TAT-P_4_-(C5)_2_ affects either. This suggests that TAT-P_4_-(C5)_2_ modulates plasticity in more subtle ways than e.g., the PKM Zeta Inhibitory Peptide (ZIP), which likewise relieves pain (Asiedu et al., 2011), but at the same time erases memories by reverting plasticity (Pastalkova et al., 2006; Wigestrand et al., 2017). Since PICK1 is involved in acute plasticity (both LTP and LTD), we have conducted our experiments with the presence of TAT-P_4_-(C5)_2_ only during the initial plasticity, not during the consolidation phase.

In conclusion, we tested the high-affinity TAT-P_4_-(C5)_2_ in the CFA model of inflammatory pain and found it to alleviate mechanical allodynia following both central (i.t) and peripheral (i.pl.) administration without affecting basal neurotransmission. We have investigated a range of potential central side effects of TAT-P_4_-(C5)_2_. Our research has looked at side effects related to general pain aspects as well as PICK1-specific possible side effects following systemic administration of TAT-P_4_-(C5)_2_ without finding any indication of side effects, high-lighting TAT-P_4_-(C5)_2_ as a promising novel lead to future treatment of chronic pain conditions. The present findings further suggest that in inflammatory pain conditions, local administration of TAT-P_4_-(C5)_2_ could have clinical translational potential as an additional means of avoiding putative central side effects not yet identified.

## Data Availability Statement

The original contributions presented in the study are included in the article, further inquiries can be directed to the corresponding author/s.

## Ethics Statement

The animal study was reviewed and approved by the Danish Animal Experimentation Inspectorate (permission number 2016-15-0201-00976).

## Author Contributions

KJ, AS, and KM conceptualized the study. KJ and GN-H conducted the experiments. KJ, KM, and AS provided funding for the study. KJ prepared data and performed the formal data analyses. KJ and KM prepared figures and wrote the manuscript. All authors contributed to the editing and review of the manuscript. All authors contributed to the article and approved the submitted version.

## Conflict of Interest

AS and KM are founders of DolorestBio, a spinout company from Copenhagen University, that have exclusive license on TAT-P_4_-(C5)_2_ and related technologies described in two patent applications filed to EPO. The remaining authors declare that the research was conducted in the absence of any commercial or financial relationships that could be construed as a potential conflict of interest.

## Publisher’s Note

All claims expressed in this article are solely those of the authors and do not necessarily represent those of their affiliated organizations, or those of the publisher, the editors and the reviewers. Any product that may be evaluated in this article, or claim that may be made by its manufacturer, is not guaranteed or endorsed by the publisher.
